# Pycnodysostosis with unusual findings: a case report

**DOI:** 10.4076/1757-1626-2-6544

**Published:** 2009-07-23

**Authors:** Quais Mujawar, Ravi Naganoor, Harsha Patil, Achyut Narayan Thobbi, Sadashiva Ukkali, Naushad Malagi

**Affiliations:** Department of Pediatrics, Al Ameen Medical CollegeAthani Road, Bijapur - 586108India

## Abstract

Pycnodysostosis is a rare clinical entity, first described in 1962 by Maroteaux and Lamy. The disease has also been named Toulouse-Lautrec syndrome, after the French artist Henri de Toulouse-Lautrec, who (it has been surmised) suffered from the disease. In 1996, the defective gene responsible for Pycnodysostosis was located, offering accurate diagnosis, carrier testing and a more thorough understanding of this disorder. It is an autosomal recessive osteochondrodysplasia, usually diagnosed at an early age with incidence estimated to be 1.7 per 1 million births. Pycnodysostosis is a lysosomal storage disease of the bone caused by a mutation in the gene that codes the enzyme cathepsin K. The syndrome has been frequently reported in history. This article reports unusual ophthalmologic findings, conductive hearing loss due to suspected otosclerosis and sandal gap deformity in a Pycnodysostosis patient.

## Case presentation

An 11-year-old Indian boy from Bijapur district of Karnataka State was referred to the paediatric unit of the Al Ameen Medical College Hospital with history of short stature, dysmorphic facies and failure to thrive for evaluation. Patient was good weight normal full term delivery at home. Maternal and Neonatal History was uneventful. Immunization was adequate with normal developmental milestones and intelligence. Parents denied any history of trauma or fractures. There was no history of frequent respiratory tract infections, snoring or tuberculosis contact. He was the youngest of three siblings born of second degree consanguineous marriage. Other siblings and parents are normal. On admission child’s weight was 28 kg. His standing height was 126 cm and upper segment was 65.5 cm and lower segment was 60.5 cm. Head circumference was 51 cm. He had mid facial hypoplasia with proptosed eyes.

He had frontal and bilateral parietal bossing .The sagittal, coronal and lambdoid sutures were separated and anterior and posterior fontanels were widely open. Examination of the mouth revealed a narrow high arched grooved palate. The teeth were hypoplastic with lateral open bite and multiple missing teeth. Bilaterally temporomandibular joints were retracted with slight deviated nasal septum to left. Scoliosis was seen. His digits were short, spoon shaped, stubby with joint laxity, metaphyseal widening and dystrophic nails. A sandal gap deformity was seen. There was no significant pallor, hepatomegaly or lymphadenopathy. Sexual maturity rating showed pre-adolescent stage. Laboratory investigations such as complete blood count, serum calcium, serum inorganic phosphate and alkaline phosphatase were normal.

The radiological findings were significant showing diffuse skeletal hyperostosis with sparing of medullary cavity. Skull X-rays showed widely separated cranial sutures and widely open anterior and posterior fontanel. The paranasal sinuses were non-pneumatised with angle of the mandible being obtuse. Acro-osteolysis was seen. The bone age was equal to his chronological age. Audiometry showed mild to moderate bilateral conductive hearing loss.

Fundus examination showed bilateral vitreous hyperplasia with lots of fibrous bands.

He was diagnosed as Pycnodysostosis based on characteristic clinical and radiological findings.

The parents of the child were offered an option for CTSK gene mutation testing for confirmation of diagnosis of pycnodysostosis, however they refused for same in view of financial constraints as the same testing is not available currently in our country (India).

We suggest for active screening of all children diagnosed with pycnodysostosis for hearing defects and ophthalmological evaluation as similar evaluation was not seen previously in published literature.

## Discussion

Pycnodysostosis is an inherited disorder of the bone caused by a mutation in the gene that codes the enzyme cathepsin K. This enzyme is important for normal bone cells called osteoclasts, to reabsorb into the bone and build new bone. The normal functioning of osteoclasts in individuals with pycnodysostosis is disrupted by a lack of cathepsin K, rendering individuals afflicted with this disorder to be unable to adequately reabsorb the component of bone called the organic matrix. This process, also called remodelling, is vital for normal bone maintenance. The bones in individuals afflicted with pycnodysostosis are abnormally dense and brittle as a result of this insufficient re-absorption process [1].

The sclerosing activity of pycnodysostosis is due to a genetic defect located on chromosome 1q21. This anomaly consists of mutations that produce mutational changes in a lysosomal cystine protease, cathepsin K, the expression of which is reduced in the osteoclasts of these patients [2]. This protease is responsible for degrading collagen type 1 that constitutes 95% of the organic bone matrix. A recent study classified the various metabolic bone diseases according to the component of the affected bone matrix. Pycnodysostosis is included in those caused by low bone remodelling [3].

Various bone diseases should be considered in the differential diagnosis of pyknodysostosis, particularly cleidocranial dysostosis, acroosteolysis, osteogenesis imperfecta, and osteopetrosis [4,5].

In cleidocranial dysostosis open fontanels and cranial sutures are also observed at an advanced age, although in this case the clavicle is also involved, a bone rarely affected in pycnodysostosis. Cleidocranial dysostosis is transmitted by autosomal dominant inheritance whereas Pycnodysostosis is autosomally recessive [5].

Bone fragility and a history of frequent fractures may suggest the possibility of diagnosing osteogenesis imperfecta, although the fractures are much more severe with other associated features like choanal atresia and blue sclera.

Clinical features of pycnodysostosis are short stature, fractures, large head with frontal and parietal bossing, open anterior fontanelle and cranial sutures, obtuse mandibular angle, prominent eyes with bluish sclerae, underdeveloped facial bones, dental anomalies, short, broad hands and feet with dystrophic nails and trunk deformities such as kyphosis, scoliosis, increased lumbar lordosis, recurrent chest infections, stridorous breathing, snoring and narrow chest. Laboratory investigations usually give results within normal limits.

Life expectancy for a Pycnodysostosis patient is normal.

Radiological findings may show some degree of widening of the distal femur. The skull shows open anterior fontanelle and sutures with small facial bones, non-pneumatised paranasal sinuses and flattened mandibular angle [6,7]. Terminal phalanges in the hand are partially or totally aplastic with loss of ungual tufts. The acromial ends of the clavicles may be aplastic. Other abnormalities include failure of complete segmentation of the atlas, axis, and the lower lumbar spine, coxa valga and abnormal radioulnar articulation.

Histologically, the appearance is similar to that of osteopetrosis but the medullary canals are present and microscopic evidence of attenuated haversian canal system is seen.

The diagnosis of pycnodysostosis is primarily based on clinical features and Radiographs; however a CTSK gene mutation analysis is the confirmatory test. Various novel mutations of cathepsin K gene in patients with pycnodysostosis have been reported in literature [8,9].

There is no specific treatment as of date for this disorder and treatment is supportive. Since bone fractures are a primary threat to those affected by Pycnodysostosis, it is important that care is taken to prevent or minimize tendencies for a fracture to occur. Such precautions include careful handling of an affected child, along with exercise and activities that are safe and do not require too much impact. Dental hygiene and regular dental checkups are especially helpful for affected individuals due to various dental anomalies [10].

## Conclusion

Our patient showed conductive hearing loss, large amount of vitreous bands and sandal gap deformity in addition to previously described features which are not reported earlier in literature. Hence we suggest for active examination of hearing capabilities and eye examination in cases of pycnodysostosis to determine if it is an isolated case or pycnodysostosis routinely presents with these features.

**Figure 1.**
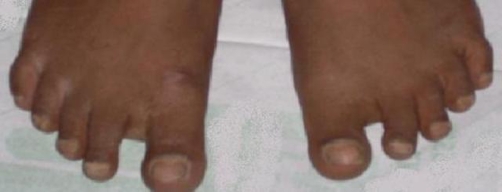
Toes showing sandal gap deformity.

**Figure 2.**
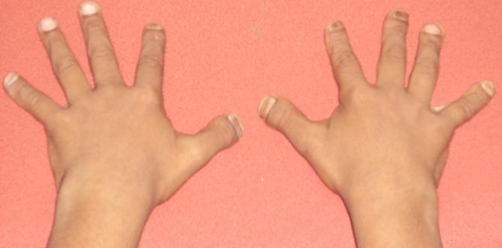
Hands showing short stubby spoon shaped digits with dystrophic nails.

**Figure 3.**
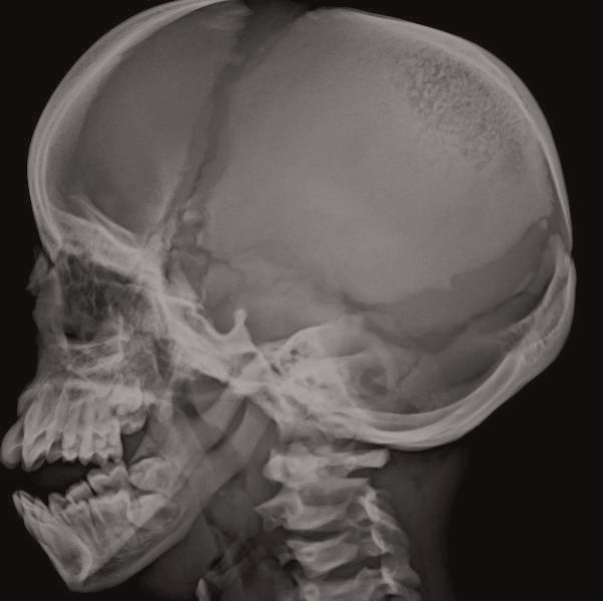
Skull X-ray showing obtuse angled mandible, open anterior and posterior fontanelle and non pneumatised sinuses.

**Figure 4.**
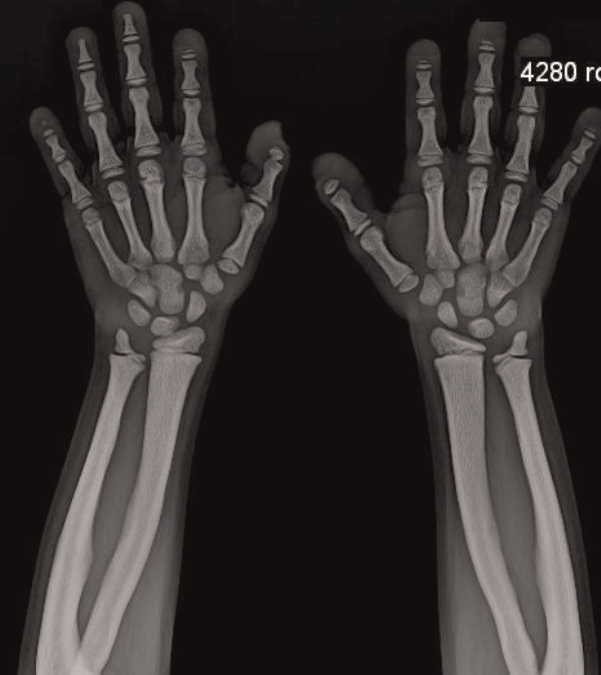
X-ray showing acroosteolysis hyperostosis with sparing of medullary cavity and normal bone age.

## Abbreviation

CTSK: Cathepsin K
